# Selective and fast oxidation of alcohol to aldehyde using novel catalytic deep eutectic solvent surfactants

**DOI:** 10.3389/fchem.2024.1416825

**Published:** 2024-10-18

**Authors:** Bahareh Shokr Chalaki, Najmedin Azizi, Zohreh Mirjafary, Hamid Saeidian

**Affiliations:** ^1^ Department of Chemistry, Science and Research Branch, Islamic Azad University, Tehran, Iran; ^2^ Chemistry and Chemical Engineering Research Centre of Iran, Tehran, Iran; ^3^ Department of Science, Payame Noor University (PNU), Tehran, Iran

**Keywords:** deep eutectic solvent-based surfactants, ferric chloride, benzyl hexadecyl dimethyl ammonium chloride, aldehyde, selective oxidation, hydrogen peroxide

## Abstract

Deep eutectic solvent (DES) has been considered as a useful catalyst and reaction medium for various organic transformations. Herein, we report the catalytic application of novel deep eutectic solvent- based surfactant (DES surfactant) for the selective and fast oxidation of alcohols to aldehydes. The readily accessible DES surfactants (FeCl_3_/BHDC) was prepared using inexpensive ferric chloride (FeCl_3_) and benzyl hexadecyl dimethyl ammonium chloride in a simple manner. The synthesized FeCl_3_/BHDC was characterized using various techniques, including, FTIR spectroscopy, thermal gravimetric analysis (TGA), scanning electron microscopy (SEM), and energy- dispersive X-ray spectroscopy (EDS) to determine its structure. The catalytic activity of FeCl_3_/BHDC in the selective oxidation of various alcohols to corresponding aldehyde derivative was investigated. The results showed the reaction could be completed within very short reaction times ranging from 2 to 15 min, while achieving good to excellent yields. This protocol offers a facile strategy and excellent efficiency in selectively oxidizing various alcohol derivatives to their respective aldehydes and ketones, utilizing hydrogen peroxide in the presence of catalytic DES surfactant.

## Introduction

Carbonyl compounds, including aldehydes and ketones, are essential in various chemical production processes in both laboratory and industrial settings (Abad et al., 2005). They serve as versatile intermediates for synthesizing a wide range of compounds with commercial applications (Gröbel and Seebach, 1977). Aldehydes, in particular, are valuable precursors for many organic transformations and find extensive use in the synthesis of agricultural and medicinal compounds (Michael and March 2007). They play a crucial role in the production of consumable goods such as perfumes, beverages, and medicinal intermediates (Patai’s Chemistry of Functional Groups, 1966). Benzaldehyde, an aromatic aldehyde, holds significant importance due to its versatile applications in various industries. Benzaldehyde serves as a primary ingredient in the production of perfumes, where its distinct almond-like fragrance is highly valued. It is also utilized in the flavor industry to provide almond or cherry-like flavors to food and beverages. Additionally, benzaldehyde acts as a precursor in the synthesis of numerous pharmaceuticals and agrochemicals, contributing to the development of agricultural and medicinal compounds (Aljaafari et al., 2022). Historically, benzaldehydes were produced through the hydrolysis of benzyl chloride or the oxidation of toluene, which had drawbacks such as the generation of chlorinated by-products and hazardous acidic compounds, as well as harsh reaction conditions and poor selectivity (Artzi et al., 2009).

In recent years, the industrial synthesis of benzaldehydes by the oxidation of benzyl alcohol has gained popularity due to its advantages of easily controllable conditions and high yield (Corey and Suggs, 1975). However, the oxidation of alcohols typically involves the use of expensive oxidizing agents like dichromate, chromic acid, or permanganate in stoichiometric quantities (Shaabani et al., 2021). These oxidants, such as potassium permanganate (KMnO_4_) and potassium dichromate (K_2_Cr_2_O_7_), possess strong oxidizing properties but also come with environmental challenges and high costs (Lou and Xu, 2002; Xu et al., 2020). To address these concerns, a potential alternative is the selective oxidation of alcohols using pure oxygen (O_2_) and hydrogen peroxide (H_2_O_2_) as the oxidants (Nagy et al., 2020). This substitution aims to minimize the environmental impact associated with the use of inorganic oxidants like KMnO_4_ and K_2_Cr_2_O_7_. Pure oxygen is readily available in the atmosphere, and hydrogen peroxide can be produced from sustainable sources or synthesized using greener methods (Nagy et al., 2020). By utilizing pure oxygen and hydrogen peroxide, the oxidation of alcohols can be performed under environmentally friendly reaction conditions, leading to improved selectivity and reduced formation of unwanted by-products (Crombie et al., 2021). Moreover, this substitution has the potential to be more cost-effective in the long run, considering the environmental and health-related costs associated with traditional inorganic oxidants (Su et al., 2010).

In recent years, there has been growing interest in exploring alternative and sustainable approaches to alcohol oxidation, including the use of catalytic system, activated surfaces (Azizi et al., 2014; Zhang et al., 2014; Liu et al., 2018; Zauche and Espenson, 1998; Li et al., 2020) and ionic liquids (Fall et al., 2010; Dai et al., 2017; Liang et al., 2010). One promising avenue is the utilization of functionalized surfaces with catalytic properties, which aligns with the principles of green chemistry (Yang et al., 2013). These surfaces can provide an alternative to traditional catalysts and offer advantages such as improved selectivity, reduced waste generation, and the potential for recyclability. Additionally, green oxidation of alcohols to aldehydes involves the use of environmentally friendly methods that minimize the use of hazardous reagents and generate less waste (Nørskov et al., 2009; Zhang et al., 2018; Tan et al., 2024; Wei et al., 2019; Wang Y. et al., 2020). These methods include aerobic oxidation using molecular oxygen as the oxidizing agent in the presence of catalysts, biocatalysis using enzymes to selectively oxidize alcohols, oxidation with hydrogen peroxide as a non-toxic oxidant, and the use of supported metal catalysts for efficient and recyclable oxidation reactions (Wei et al., 2022; Wang J. et al., 2020; Li et al., 2022; Wang F. et al., 2024).

DES are novel environmentally friendly solvents that have gained noteworthy attention in recent years due to their various applications in chemistry, materials science, and biotechnology (Zhang et al., 2012). DES are formed by the mixing of two or more components, which undergo a eutectic reaction to form a liquid phase at relatively low temperatures (Dai et al., 2013). DES offer several advantageous properties that make them appealing as solvents and reaction media in various applications (Francisco et al., 2013). These include their low volatility, resulting in reduced release of volatile organic compounds and improved safety during handling; low toxicity, making them environmentally friendly alternatives to conventional solvents; high thermal stability, enabling their use at elevated temperatures without decomposition; wide liquid range, providing flexibility in their application across different temperature ranges; and versatility, as DES can be tailored by selecting different combinations of starting materials, allowing for a wide range of solvents with tunable properties (Wagle et al., 2013; Tang and Row, 2013; Mbous et al., 2017; Abo-Hamad et al., 2015; Liu et al., 2015). These characteristics make DES promising candidates for sustainable and efficient solvent systems in fields such as chemistry, materials science, and biotechnology (Li J. et al., 2023; Procopio and Ramón, 2024; Paparella et al., 2024; Li X. et al., 2023; Cicco et al., 2022). Furthermore, the versatility, tunability, and environmentally friendly nature of metal salts based DES make them valuable solvents and catalyst with diverse applications across multiple fields (Latos et al., 2024; Zhang et al., 2019; Rodríguez-Álvarez et al., 2023; Matuszek et al., 2016; Estager et al., 2014).

Surfactants, on the other hand, are amphiphilic molecules that contain both hydrophilic and hydrophobic sites. They are widely used in various industries and applications, including emulsification, detergency, and solubility. The combination of deep eutectic solvents and surfactants has led to the development of deep eutectic solvent surfactants (Basu et al., 2023). DES surfactants are surfactants that incorporate DES as part of their molecular structure or as the solvent medium in which they are dispersed (Lakshmipraba et al., 2022). DES surfactants offer several advantages compared to conventional surfactants. Firstly, the use of DESs as the solvent medium provides enhanced solubility and stability for the surfactant molecules. This leads to improved performance in applications such as emulsification, dispersion, and solubility. Secondly, DES surfactants exhibit unique properties based on the specific combination of the DES and surfactant components (Manasi et al., 2023). For example, the choice of HBA and HBD in the deep eutectic solvent can influence the polarity and hydrophilic-lipophilic balance of the DES surfactants. This allows for the tailoring of surfactant properties to meet specific application requirements (Munive-Olarte et al., 2022).Additionally, they provide a greener alternative to conventional surfactants. The use of DESs as the solvent medium reduces the reliance on volatile organic compounds and toxic solvents, contributing to improved environmental sustainability (Sheikh Asadi et al., 2024).

**FIGURE 1:**
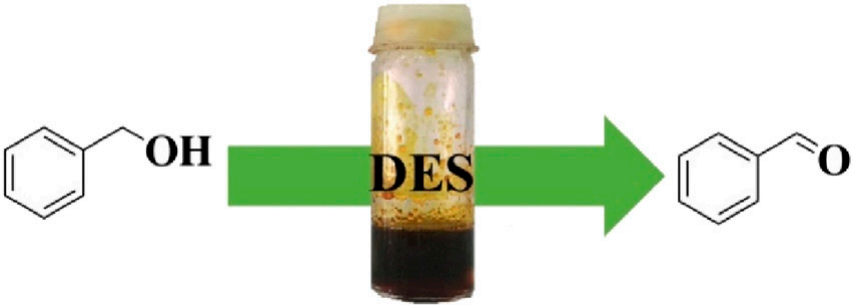
The selective oxidation of alcohols via FeCl_3_/BHDC system.

As part of our ongoing research on developing efficient homogeneous catalytic systems (Seyyed Shahabi et al., 2020; Azizi and Edrisi, 2017; Mirmashhori et al., 2006) and enhancing the properties of catalytic frameworks using DESs for various transformations, we have developed a new DES surfactant that was formed by chemically combining FeCl_3_ with BHDC (Figure 1). In addition, their catalytic efficiency for selective oxidation of alcohols to their corresponding carbonyl compounds was also investigated.

## Experimental section

### Materials and methods

All chemicals used in the experiments were commercially available and were obtained from chemical supplier without further purification. Thin-layer chromatography (TLC) was employed to monitor the reactions under UV light, utilizing Merck 60 HF254 silica plates. The melting points were determined using Büchi 535 melting point apparatus. SEM images were captured using a ZEISS scanning electron microscope. TGA was conducted on an STA-1500 instrument at a heating rate of 10°C/min in air atmosphere. The FT-IR spectra was recorded using a Bomem MB-Series FT-IR spectrometer.

### Preparation of DES

**FIGURE 2:**
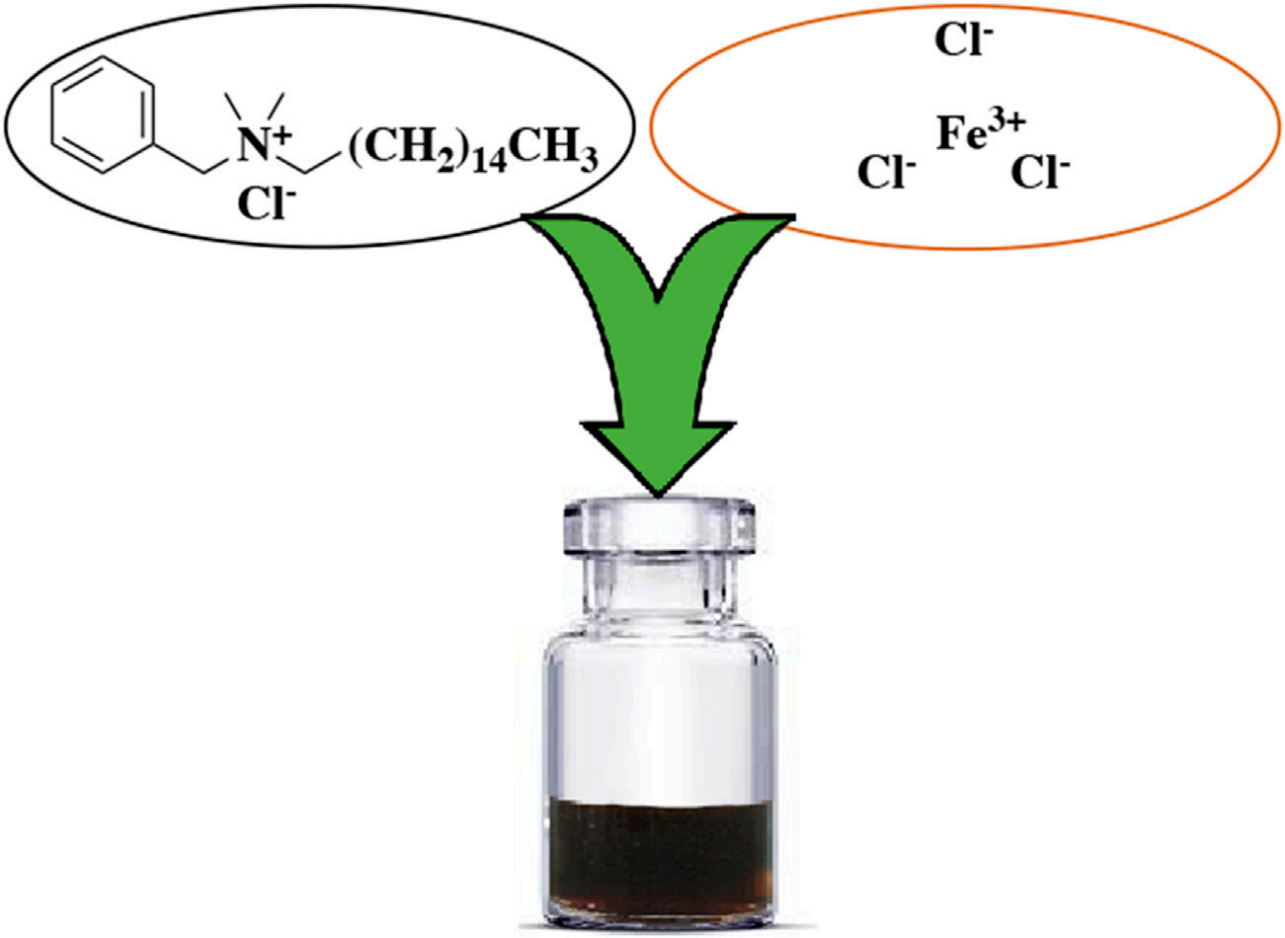
FeCl_3_/BHDC preparation.

FeCl_3_.6H_2_O (200 mmol) and BHDC (100 mmol) were mixed together in a round-bottom flask. The resulting mixture was then heated at 60 °C for a 20 min. After the heating process, a dark brown liquid was obtained without undergoing any further purification steps (Figure 2).

### General procedure

An alcohol (1.00 mmol) and FeCl_3_/BHDC (10 mg) were mixed in a test tube. Subsequently, H_2_O_2_ 32% (3.00 mmol) was added dropwise to the mixture. The reaction was observed to be exothermic and completed within 2–15 min at room temperature, as visually monitored. After the reaction was completed, water (2 mL) and ethyl acetate (2 mL) was added, and the ethyl acetate layer was separated and rapidly purified with silica gel flash chromatography to get the desired aldehyde product. The conversion and selectivity of the reaction were determined by GC analysis. All compounds were known and were characterized by melting and boiling points found to be identical with the ones described in the literature.

## Results and discussion

The FeCl_3_/BHDC was prepared using a simple and straightforward method, as depicted in Figure 2. The prepared FeCl_3_/BHDC was subsequently subjected to characterization using various spectroscopic techniques. The spectroscopic analysis provides valuable information about the composition, bonding, and functional groups present in the FeCl_3_/BHDC, enabling a comprehensive understanding of its molecular structure and properties.

**FIGURE 3:**
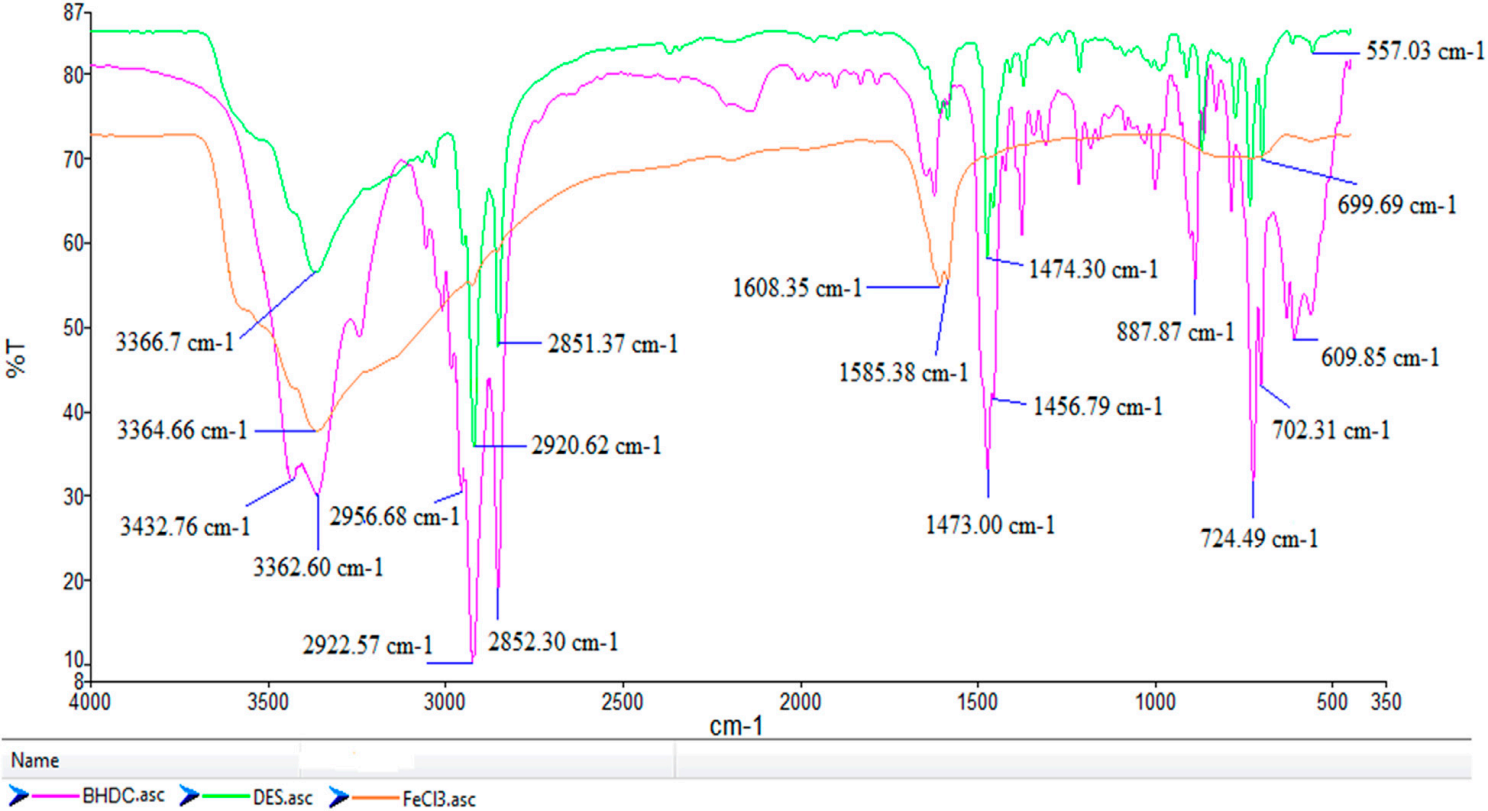
FT-IR spectra for DES, FeCl_3_, BHDC.

The FT-IR spectroscopy analysis of FeCl_3_/BHDC, FeCl_3_.6H_2_O and BHDC were depicted in Figure 3 showed characteristic peaks and changes in specific frequency regions. In the FT-IR spectrum of FeCl_3_/BHDC, a broad peak was observed in the range of 3432–3352 cm^−1^, which can be attributed to the presence of water in FeCl_3_.6H_2_O.The strong bands in the range of 2,852–2,922 cm^−1^ correspond to the C-H stretching vibrations of the CH_2_ group. In the lower frequency region, two peaks were observed around 1,473 cm^−1^, corresponding to stretching vibrations of benzene rings, and a peak at 1,349 cm^−1^ was observed, which corresponds to the stretching vibrations of the N–CH_2_ further confirming the presence of alkyl chains in BHDC. In the region of 609 cm^−1^ and 562 cm^−1^, peaks were observed that can be attributed to the bending vibrations of Fe-Cl bonds. The main changes were observed in the 350–700 cm^−1^ region, which is associated with the interaction between FeCl_3_ and BHDC. These peaks in this region can indicate the formation of coordination complexes in the molecular structure.

**FIGURE 4:**
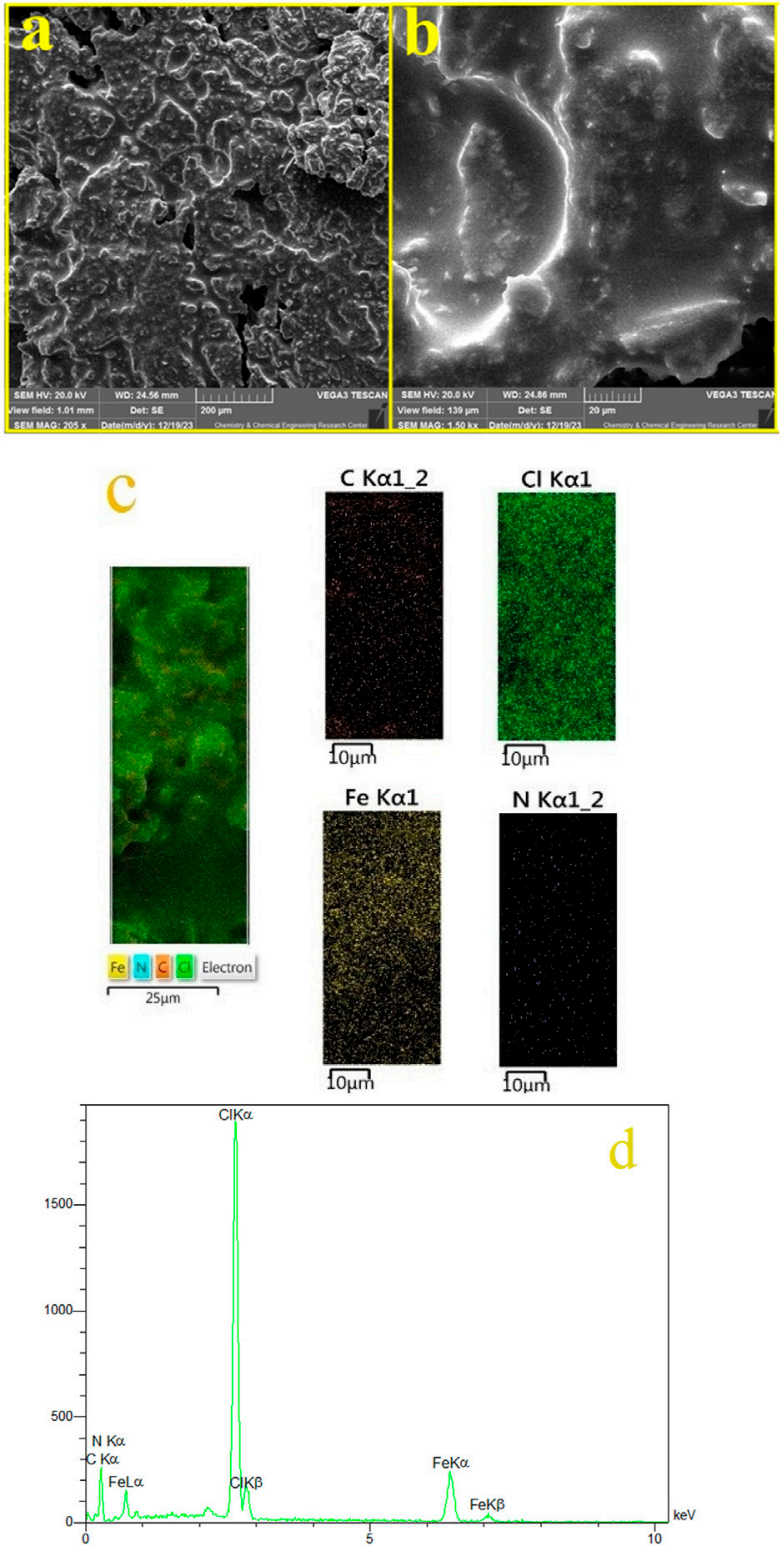
Sem, EDX and EDX-mapping of the FeCl_3_/BHDC. **(A, B)** SEM, **(C)** EDX-Mapping, **(D)** EDX.

Figure 4 showcases the SEM images of the FeCl_3_/BHDC. The images reveal that the structure of the FeCl_3_/BHDC exhibits a plate-like morphology. This observation suggests that the FeCl_3_/BHDC possesses a layered or sheet-like structure, with distinct flat or plate-like particles. The chemical composition of the FeCl_3_/BHDC was analyzed using EDS, and the corresponding results are presented in Figure 4B. The EDS analysis revealed the presence of carbon, iron, chlorine, and nitrogen in the structure of the FeCl_3_/BHDC. These findings provide additional confirmation of the successful reaction between FeCl_3_ and BHDC, as the detected elements correspond to the components used in the synthesis of the FeCl_3_/BHDC. In addition to the EDS analysis, EDS mapping was also performed to investigate the elemental distribution within the FeCl_3_/BHDC (Figure 4C). The results of the EDS mapping analysis demonstrated a uniform distribution of elements throughout the DES structure. This means that the elements present in the DESS, such as carbon (C), iron (Fe), chlorine (Cl), and nitrogen (N), were evenly dispersed and distributed across the entire sample.

**FIGURE 5:**
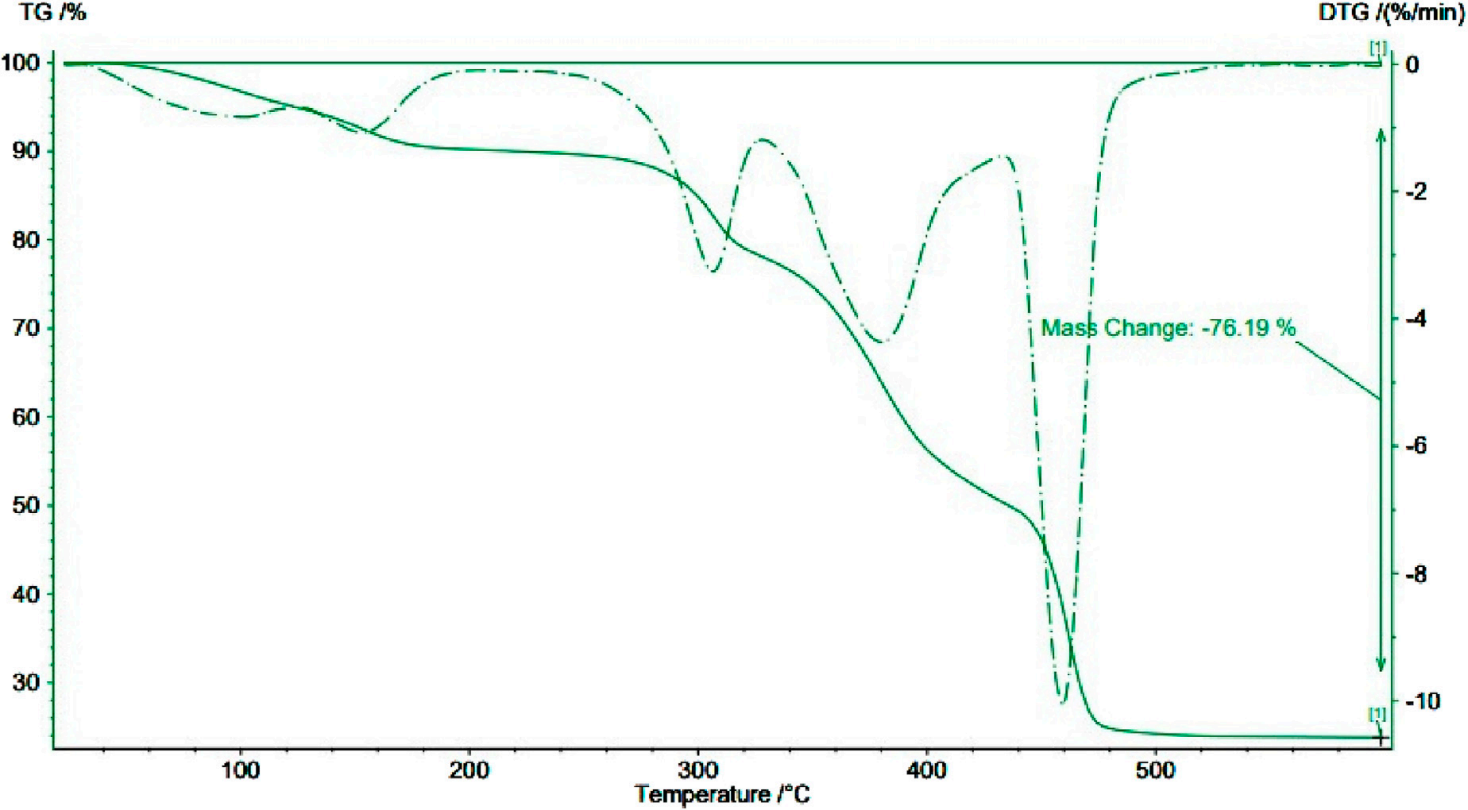
TGA curves of FeCl_3_/BHDC.

The thermal stability of FeCl_3_/BHDC was investigated using thermogravimetric analysis under air conditions. The results of the TGA analysis are depicted in Figure 5, which reveals four distinct mass losses occurring at different temperature ranges. The first mass loss occurs below 120 °C and is attributed to the evaporation of water molecules weakly bound to FeCl_3_.6H_2_O through hydration. The second mass loss takes place in the temperature range of 120°C–180°C corresponds to the loss of water molecules that are strongly linked (dehydrated water). The third step, observed between 250°C and 350°C, corresponds to the decomposition of BHDC. Finally, the fourth mass loss occurs between 350°C and 500°C and corresponds to the complete decomposition of anhydrous ferric chloride with the detachment of HCl (g) and Cl_2_ (g) gases. This transformation leads to the formation of iron oxide. From the TGA analysis presented in Figure 5, it is evident that the synthesized FeCl_3_/BHDC exhibits higher thermal stability compared to the BHDC surfactant (Müller et al., 2014; Ahmad Wagay et al., 2020).

After preparation and characterization of FeCl_3_/BHDC, their catalytic activity in the oxidation process of benzyl alcohol was evaluated. The efficiency of the catalyst, as well as the influence of the oxidant, and reaction time, were optimized. Table 1 presents the results of the reaction conducted with varying amounts of FeCl_3_/BHDC catalyst (5 mg, 7 mg, 10 mg, 15, and 20 mg). Based on the results presented in Table 1, it was determined that the optimal amount of FeCl_3_/BHDC catalyst for the alcohol oxidation process was 10 mg as it gives higher yield (98%) of the desired product and shorter reaction time compared to other catalyst loadings tested.

**TABLE 1:** Optimizing the amount of FeCl_3_/BHDC.

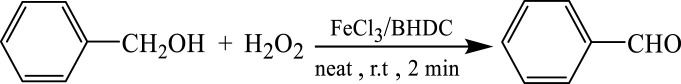					
Entry	Alcohol		H_2_O_2_	DES (mg)	Yield (%)
1	Benzyl Alcohol	1 mmol	3 mmol	5	42
2	Benzyl Alcohol	1 mmol	3 mmol	7	70
3	Benzyl Alcohol	1 mmol	3 mmol	10	98
4	Benzyl Alcohol	1 mmol	3 mmol	15	98
5	Benzyl Alcohol	1 mmol	3 mmol	20	98

Furthermore, as shown in Table 2, the catalytic activity of FeCl_3_/BHDC was compared with FeCl_3_ (Entry 7) and BHDC (Entry 8) alone to evaluate the efficiency of the desired FeCl_3_/BHDC in the oxidation process. It was found that the combined FeCl_3_/BHDC gives the highest value for yield (98%). Next, different quantities of the oxidant were tested. Based on the results, it was determined that 3 mmol of H_2_O_2_ was the optimal quantity of oxidant for the oxidation process. The effect of different solvents on the reaction yields was also investigated (Table 2, entries 10–13). The tested solvents were water, acetonitrile, ethyl acetate, and methanol. After conducting these studies, it was determined that the solvent-free condition was the most favorable condition for the reaction. Based on the overall analysis, it was concluded that the most favorable conditions for the model reaction were achieved by using 10 mg of FeCl_3_/BHDC catalyst at room temperature, in the presence of 3 mmol of H_2_O_2_ as the oxidant, and under solvent-free conditions. Based on entry 9, the reaction without catalyst and solvent was failed.

**TABLE 2:** Optimization parameters in the model reaction.

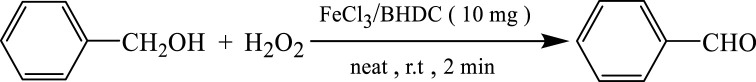				
Entry	FeCl_3_/BHDC	Solvent	H_2_O_2_ (mmol)	Yield (%)^a^
1	FeCl_3_/BHDC	—	0.50	45
2	FeCl_3_/BHDC	—	1.00	70
3	FeCl_3_/BHDC	—	1.50	85
4	FeCl_3_/BHDC	—	2.00	90
5	FeCl_3_/BHDC	—	2.50	95
6	FeCl_3_/BHDC	—	3.00	98
7	FeCl_3_	—	3.00	60
8	BHDC	—	3.00	10
9	—	—	3.00	0
10	FeCl_3_/BHDC	H_2_O	3.00	60
11	FeCl_3_/BHDC	EtOAc	3.00	42
12	FeCl_3_/BHDC	MeOH	3.00	45
13	FeCl_3_/BHDC	Acetonitrile	3.00	50

^a^ Conversion determined by GC, analysis.

Table 3 demonstrates the results of the oxidation of various alcohols to their corresponding carbonyl compounds under the optimized conditions. The findings indicate that all the products were obtained in high yields, justifying the effectiveness of the catalytic system. Table 3 displays using a range of benzylic alcohol derivatives, including primary and secondary benzylic alcohols, which were subjected to oxidation. Secondary benzylic alcohols exhibited higher reactivity in this catalytic system, as evidenced by shorter reaction times and higher yields compared to other compounds. The reactivity of primary benzylic alcohols was influenced by the nature of the substituent group; Primary benzylic alcohols containing an electron-withdrawing group demonstrated superior performance, achieving higher yields compared to those containing an electron-donating group.

**TABLE 3:** Oxidation of alcohols with FeCl_3_/BHDC as a catalyst^a^.

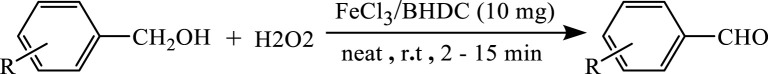			
Entry	Reagent	Time (min)	Conversion^b^(%)
1	Benzyl Alcohol	2	98
2	2-F-Benzyl Alcohol	2	92
3	3- NO_2_-Benzyl Alcohol	2	95
4	4- NO_2_-Benzyl Alcohol	2	97
5	2- NO_2_-Benzyl Alcohol	5	97
6	4-tert-Benzyl Alcohol	7	95
7	1-Phenylethanol	9	97
8	Benzhydrol	15	90
9	2-Cl-Benzyl Alcohol	10	91
10	4-Cl-Benzyl Alcohol	10	95
11	4-Br-Benzyl Alcohol	8	97
12	4-OMe-Benzyl Alcohol	15	92
13	4-Me-Benzyl Alcohol	15	90
14	2-Pyridinemethanol	2	97
15	4-Pyridinemethanol	2	97

^a^ Reaction condition: Alcohols (1.0 mmol), FeCl_3_/BHDC (10 mg), room temperature, H_2_O_2_ (3.0 mmol) and under solvent free condition.

^b^ Conversion determined by Gas chromatography analysis.

In comparison with other oxidation systems reported in the literature, our proposed method is relatively more facile and favorable having shorter reaction time and higher yield (Table 4).

**TABLE 4:** Comparison of the performance of various catalyst systems and methods in the synthesis of benzyl alcohol.

Entry	Cat	Oxidant	Solvent	Temp (°C)	Time (h)	Yield (%)	Ref.
1	CoFe_2_O_4_ (2 mg)	O_2_	DMA	150	24	96.7	Gao et al. (2023)
2	Au/Al_2_O_3_ (100 mg)	O_2_	neat	130	5	44.8	Choudhary et al. (2005)
3	CeO_2_ (300 mg)	H_2_O_2_	neat	50	6	68	Tamizhdurai et al. (2017)
4	Au/γ-Al_2_O_3_ (75 mg)	TBHP	neat	125	5	73.4	Ndolomingo and Meijboom (2017)
5	CrBO_3_ (500 mg)	O_2_	neat	100	4	41	Ozturk et al. (2008)
6	NiFe_2_O_4_ NPs (10 mg)	TBHP	Acetonitrile	60	3	85	Iraqui et al. (2020)
7	CoAl_2_O_4_ (500 mg)	H_2_O_2_	Acetonitrile	80	5	61.5	Ragupathi et al. (2015)
8	MgAl_2_O_4_@ SiO_2_–PTA (50 mg)	H_2_O_2_	H_2_O	90	1.5	96	Heravi et al. (2020)
9	Au/γ-Al_2_O_3_ (300 mg)	H_2_O_2_	H_2_O	80	2	98	Long and Quan (2015)
10	[C_7_H_7_N(CH_3_)_3_]_9_PW_9_O_34_ (100 mg)	H_2_O_2_	DMAc	80	0.5	95	Weng et al. (2008)
11	Pd/Al-PILC (52 mg)	H_2_O_2_	Acetonitrile	82	3	82	Trikittiwong et al. (2023)
12	CoFe_2_O_4_ (5 mg)	O_2_	DMSO	25	14	95	Changwal and Ameta (2024)
13	Pd/CeO_2_ (20 mg)	O_2_	neat	120	2	90	Wang et al. (2022)
14	Pd/eg-C_3_N_4_-AN (20 mg)	O_2_	neat	90	8	81.8	Li et al. (2023c)
15	MnFe_2_O_4_ (20 mg)	H_2_O_2_	Acetonitrile	80	3	73	Wang et al. (2024b)
16	FeCl_3_/BHDC (10 mg)	H_2_O_2_	neat	25	2 min	98	This work

**FIGURE 6:**
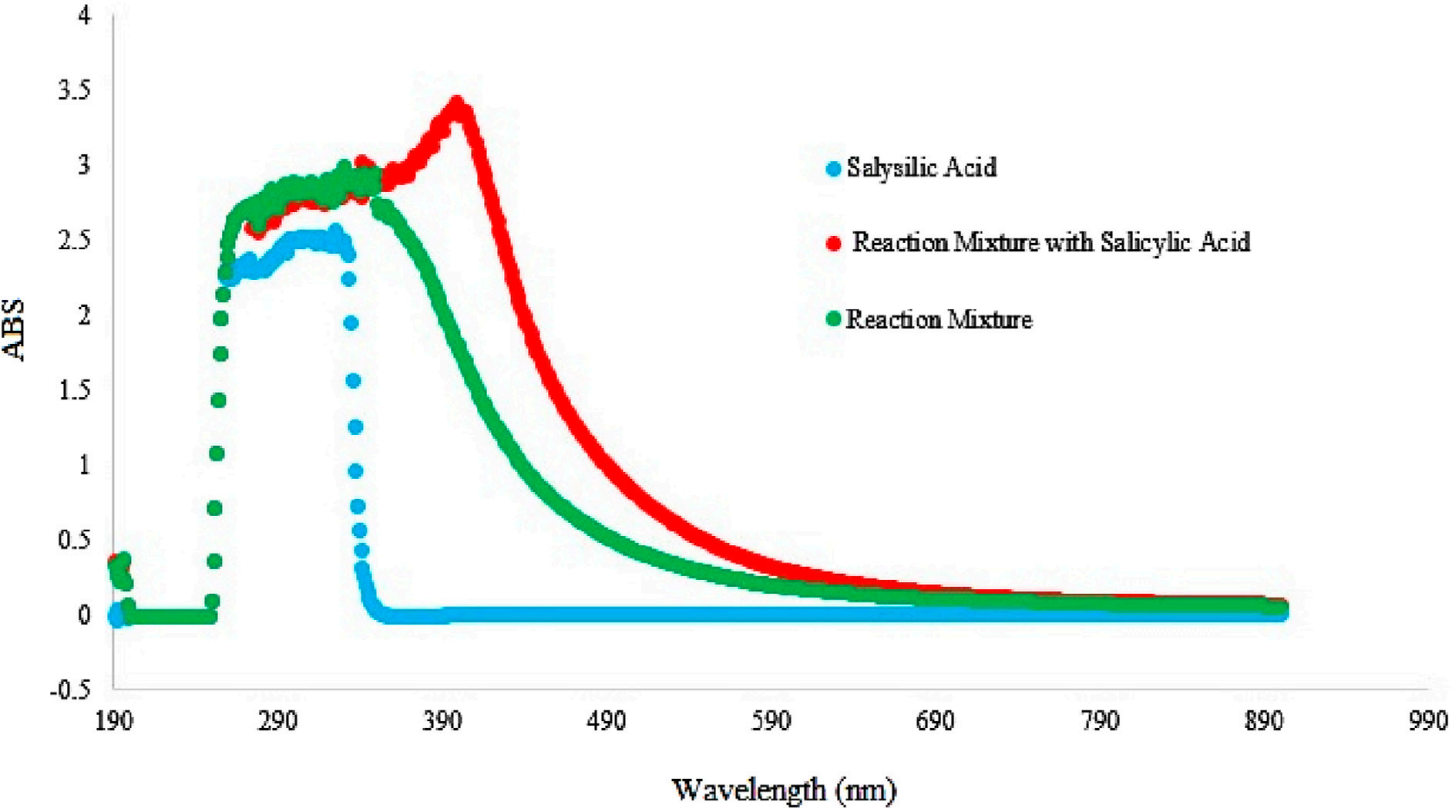
Hydroxyl radical capture in the presence of salicylic acid with UV-Vis detection.

To investigate the formation of hydroxyl radicals during the reaction process, a verification experiment was conducted using salicylic acid as a hydroxyl radical trap. The results, shown in Figure 6, revealed a distinct adsorption peak in the UV-vis spectra at approximately 290–590 nm, with a maximum absorption at 470 nm. This peak corresponds to the characteristic absorption of adducts formed between hydroxyl radicals and salicylic acid. These findings strongly indicate the presence of hydroxyl radicals during the oxidation, confirming their involvement in the oxidation of alcohols to aldehydes (Chen et al., 2023; Li W. et al., 2023).

**FIGURE 7:**
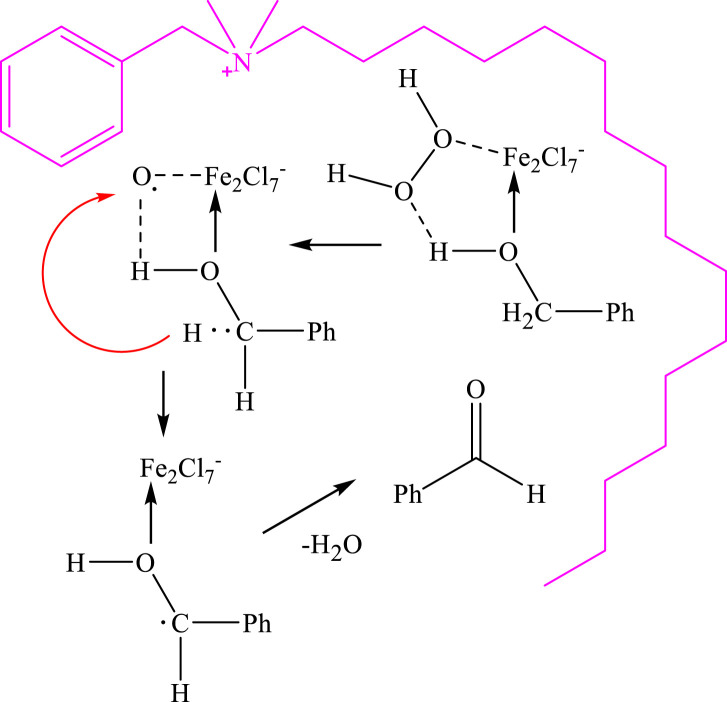
Possible oxidation mechanisms of the reaction.

The proposed reaction mechanism for the oxidation of alcohols in the presence of DES is depicted in Figure 7. DES plays two main roles in this system. Firstly, due to the water insolubility of benzyl alcohols and the surface activity of DES, it enables successful biphasic catalysis between the aqueous and alcohol phases for the oxidation reaction. Secondly, iron salts in DES acts as a catalyst on the interphase by coordinating with both the alcohols and H_2_O_2_ to form an iron complex. The coordinated intermediate then undergoes a reaction with the alcohol, leading to the oxidation of the alcohol and the formation of the aldehyde product.

**FIGURE 8:**
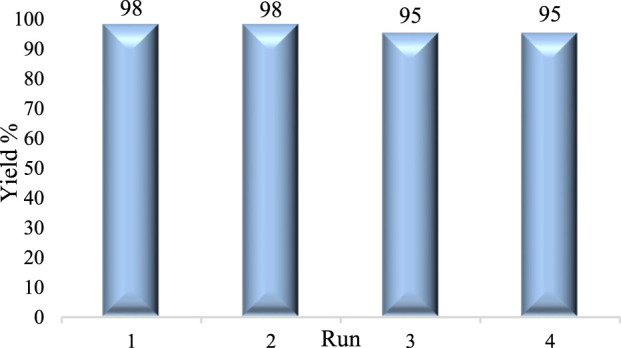
Reusability run of DES.

The recycling potential of the catalyst is crucial for industrial applications as it can significantly reduce costs and waste generation. The stability and reusability of the DES catalyst were investigated, and the results are presented in Figure 8. The model reaction benzyl alcohols in the presence of FeCl_3_/BHDC and H_2_O_2_ as the catalyst system was employed. After the completion of the reaction, water (2 mL) and ethyl acetate (2 mL) were added, and two phases were separated. The water phase, containing the DES catalyst, was then subjected to evaporation under reduced pressure. The remaining DES was retained and utilized for subsequent consecutive runs. The results indicated that the DES catalyst system exhibited reusability for up to four consecutive runs without any significant decrease in yields.

## Conclusion

In our study, we have successfully synthesized and characterized deep eutectic solvent surfactants (FeCl_3_/BHDC) using easily accessible starting materials. This synthetic method offers simplicity and accessibility, making it practical for large-scale production. The applications of FeCl_3_/BHDC was investigated as a catalyst for selective oxidation reactions using aqueous hydrogen peroxide. FeCl_3_/BHDC exhibited excellent catalytic performance, enabling the conversion of various primary and secondary alcohol derivatives into their corresponding aldehydes and ketones with higher yields and shorter reaction time. These approaches contribute to sustainable and greener chemical synthesis by reducing environmental impacts and promoting the use of renewable and non-toxic reagents.

## Data Availability

The original contributions presented in the study are included in the article/Supplementary Material, further inquiries can be directed to the corresponding author.
