# Supplementary material for: Selective and fast oxidation of alcohol to aldehyde using novel catalytic deep eutectic solvent surfactants
Source: Front Chem. 2024 Oct 18;12:1416825. doi: 10.3389/fchem.2024.1416825 (PMC11527706; doi:10.3389/fchem.2024.1416825)
Supplement: Supplementary file 1 [file DataSheet1.PDF]

# Supporting Information

Selective and Fast Oxidation of Alcohol to aldehyde Using Catalytic Novel Deep Eutectic Solvent Surfactants

**Bahareh Shokr Chalaki,[a] Najmedin Azizi,*[a] Zohreh Mirjafary,[b] Hamid Saeidian,[c]**

[a] *Department of Chemistry, Science and Research Branch. Islamic Azad University, P.O. Box 14515/775, Tehran, Iran.*

[b] *Chemistry & Chemical Engineering Research Center of Iran, P.O. Box 14335 186, Tehran, Iran.*

[c] *Department of Science, Payame Noor University (PNU), PO Box 19395 4697, Tehran, Iran.*

Table of Contents

GC …………………………………………………………………………………………… 3-11

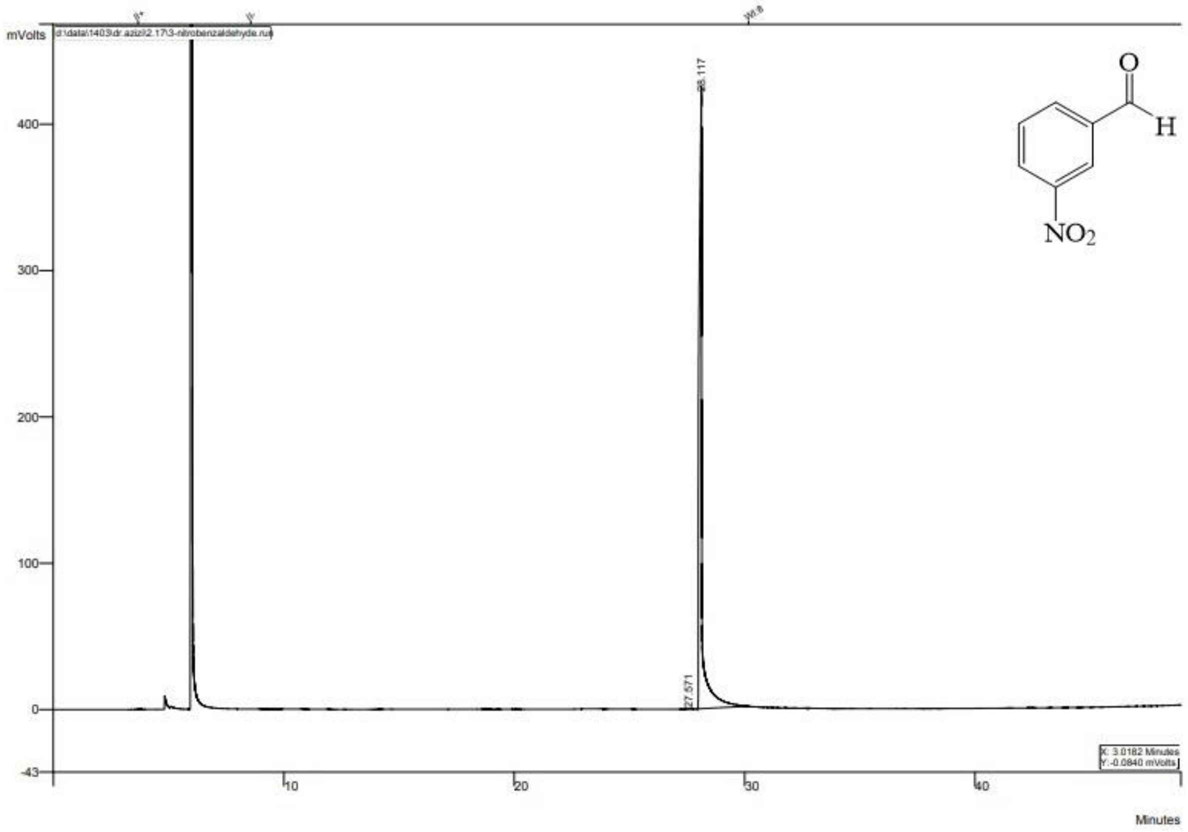


```
Title        :
Run File     : d:\data\1403\dr.azizi\2.17\3-nitrobenzaldehyde.run
Method File  : 3-nitrobenzaldehyde-middle.mth
Sample ID    : 3-nitrobenzaldehyde

Injection Date: 5/7/2024 02:31 Þ.Ù    Calculation Date: 6/4/2002 05:39 Þ.Ù

Operator    :                         Detector Type: 3800 (100 Volts)
Workstation:                          Bus Address  : 44
Instrument : Varian Star #1           Sample Rate  : 10.00 Hz
Channel    : Middle = FID             Run Time     : 48.950 min

** GC Workstation Version 6.20 ** 03154-6390-826-0630 **

Run Mode          : Analysis
Peak Measurement: Peak Area
Calculation Type: Percent
```

| Peak No. | Peak Name | Result () | Ret. Time (min) | Time Offset (min) | Area (counts) | Sep. Code | Width 1/2 (sec) | Status Codes |
|---|---|---|---|---|---|---|---|---|
| 1 | | 0.0688 | 27.571 | 0.000 | 2652 | BB | 0.0 | |
| 2 | | 99.9312 | 28.117 | 0.000 | 3853216 | VB | 8.3 | |
| | Totals: | 100.0000 | | 0.000 | 3855868 | | | |

```
Total Unidentified Counts :    3855868 counts

Detected Peaks: 10          Rejected Peaks: 8      Identified Peaks: 0

Multiplier: 1           Divisor: 1          Unidentified Peak Factor: 0

Baseline Offset: -79 microVolts         LSB:     1 microVolts

Noise (used): 31 microVolts - monitored before this run

Manual injection
```

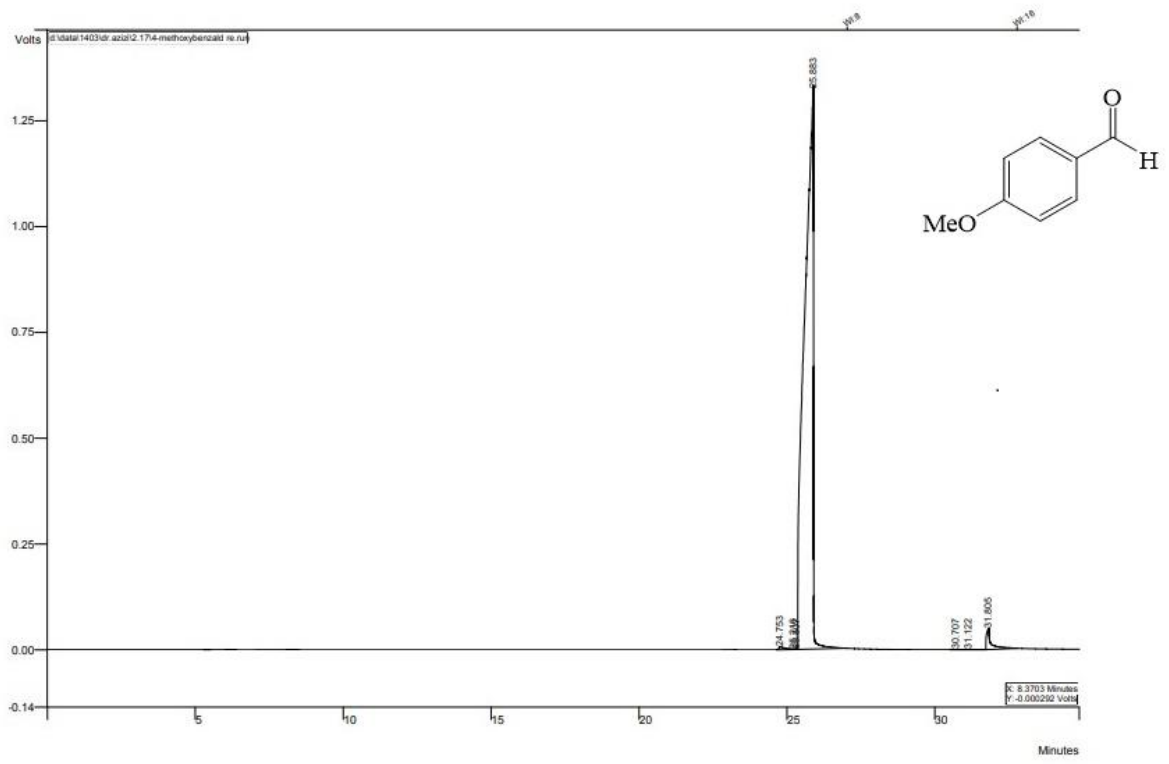


```
Title       :
Run File    : d:\data\1403\dr.azizi\2.17\4-methoxybenzald re.run
Method File : 4-methoxybenzald re-middle.mth
Sample ID   : 4-methoxybenzald re

Injection Date: 5/7/2024 09:37 È.Ù    Calculation Date: 6/4/2002 05:40 Þ.Ù

Operator    :                        Detector Type: 3800 (100 Volts)
Workstation:                         Bus Address  : 44
Instrument  : Varian Star #1         Sample Rate  : 10.00 Hz
Channel     : Middle = FID           Run Time     : 34.890 min

** GC Workstation Version 6.20 ** 03154-6390-826-0630 **

Run Mode         : Analysis
Peak Measurement: Peak Area
Calculation Type: Percent
```

| Peak No. | Peak Name | Result () | Ret. Time (min) | Time Offset (min) | Area (counts) | Sep. Code | Width 1/2 (sec) | Status Codes |
|---|---|---|---|---|---|---|---|---|
| 1 | | 0.3792 | 24.753 | 0.000 | 106965 | BV | 16.3 | |
| 2 | | 0.0182 | 25.216 | 0.000 | 5141 | VV | 0.0 | |
| 3 | | 0.0055 | 25.307 | 0.000 | 1564 | VV | 0.0 | |
| 4 | | 97.4458 | 25.883 | 0.000 | 27490866 | VB | 14.5 | |
| 5 | | 0.0544 | 30.707 | 0.000 | 15360 | BV | 19.9 | |
| 6 | | 0.0932 | 31.122 | 0.000 | 26282 | VP | 28.2 | |
| 7 | | 2.0037 | 31.805 | 0.000 | 565278 | PB | 7.0 | |
| | Totals: | 100.0000 | | 0.000 | 28211456 | | | |

```
Total Unidentified Counts :   28211456 counts

Detected Peaks: 10          Rejected Peaks: 3       Identified Peaks: 0

Multiplier: 1            Divisor: 1          Unidentified Peak Factor: 0

Baseline Offset: -52 microVolts           LSB:     1 microVolts

Noise (used): 32 microVolts - monitored before this run

Manual injection
```

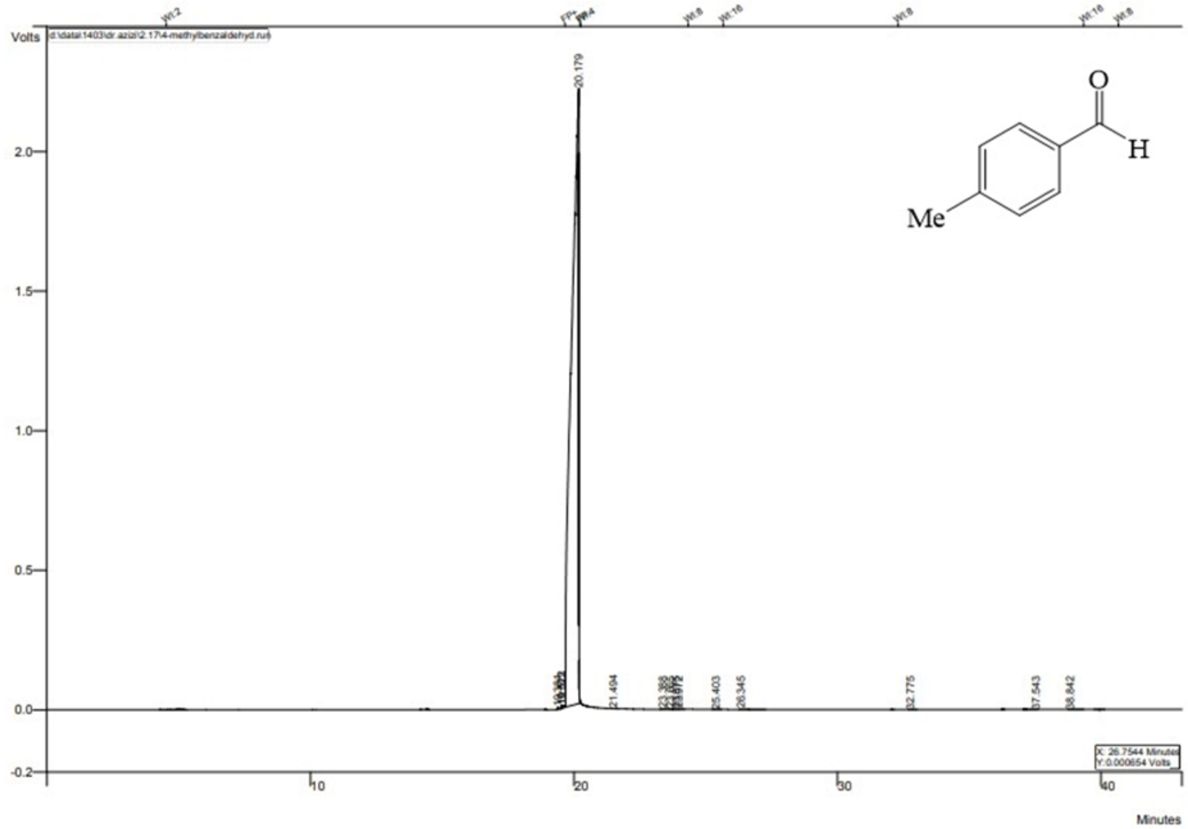


```
Title         :
Run File      : d:\data\1403\dr.azizi\2.17\4-methylbenzaldehyd.run
Method File   : 4-methylbenzaldehyd-middle.mth
Sample ID     : 4-methylbenzaldehyd

Injection Date: 5/7/2024 12:40 Þ.Ù    Calculation Date: 6/4/2002 05:43 Þ.Ù

Operator     :                        Detector Type: 3800 (100 Volts)
Workstation:                          Bus Address   : 44
Instrument : Varian Star #1           Sample Rate   : 10.00 Hz
Channel     : Middle = FID            Run Time      : 43.078 min

** GC Workstation Version 6.20 ** 03154-6390-826-0630 **

Run Mode          : Analysis
Peak Measurement: Peak Area
Calculation Type: Percent
```

| Peak No. | Peak Name | Result () | Ret. Time (min) | Time Offset (min) | Area (counts) | Sep. Code | Width 1/2 (sec) | Status Codes |
|---|---|---|---|---|---|---|---|---|
| 1 | | 0.0827 | 19.381 | 0.000 | 34944 | BP | 7.1 | |
| 2 | | 0.0776 | 19.522 | 0.000 | 32775 | PV | 2.1 | |
| 3 | | 0.0948 | 19.573 | 0.000 | 40050 | VB | 0.0 | |
| 4 | | 99.5663 | 20.179 | 0.000 | 42067636 | BB | 20.6 | |
| 5 | | 0.0049 | 21.494 | 0.000 | 2058 | BB | 3.0 | |
| 6 | | 0.0731 | 23.388 | 0.000 | 30864 | BV | 13.5 | |
| 7 | | 0.0256 | 23.665 | 0.000 | 10815 | VV | 13.5 | |
| 8 | | 0.0080 | 23.875 | 0.000 | 3385 | VV | 13.5 | |
| 9 | | 0.0081 | 23.972 | 0.000 | 3403 | VB | 55.1 | |
| 10 | | 0.0071 | 25.403 | 0.000 | 3011 | BB | 12.9 | |
| 11 | | 0.0345 | 26.345 | 0.000 | 14593 | BB | 11.7 | |
| 12 | | 0.0024 | 32.775 | 0.000 | 1035 | BB | 0.0 | |
| 13 | | 0.0035 | 37.543 | 0.000 | 1484 | BB | 0.0 | |
| 14 | | 0.0114 | 38.842 | 0.000 | 4814 | BB | 13.5 | |
| | Totals: | 100.0000 | | 0.000 | 42250867 | | | |

```
Total Unidentified Counts :   42250868 counts

Detected Peaks: 22          Rejected Peaks: 8       Identified Peaks: 0

Multiplier: 1           Divisor: 1          Unidentified Peak Factor: 0

Baseline Offset: -51 microVolts           LSB:     1 microVolts

Noise (used): 36 microVolts - monitored before this run

Manual injection
```

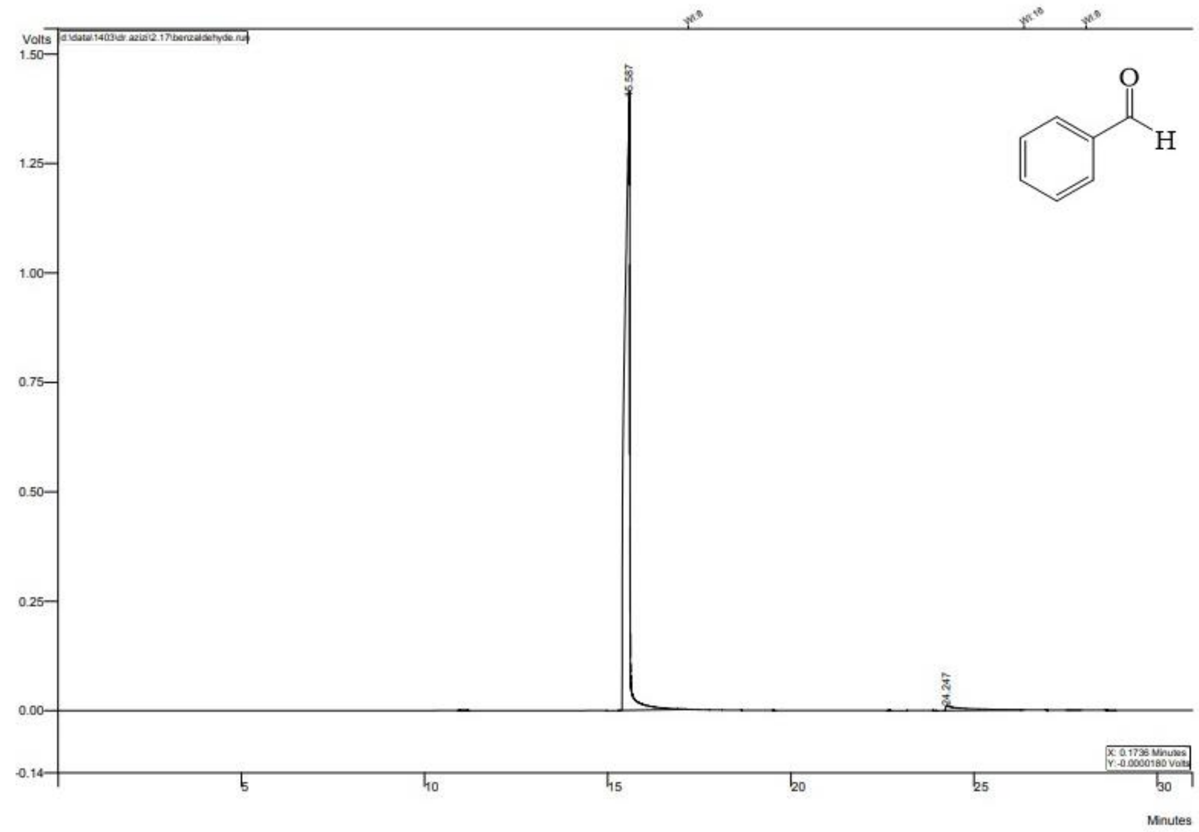


Title        :
Run File     : d:\data\1403\dr.azizi\2.17\benzaldehyde.run
Method File  : 4-methoxybenzald re-middle.mth
Sample ID    : benzaldehyde

Injection Date: 5/6/2024 11:21 È.Ù    Calculation Date: 6/4/2002 05:40 Þ.Ù

Operator    :
Workstation:
Instrument  : Varian Star #1
Channel     : Middle = FID

Detector Type: 3800 (100 Volts)
Bus Address  : 44
Sample Rate  : 10.00 Hz
Run Time     : 30.967 min

** GC Workstation Version 6.20 ** 03154-6390-826-0630 **

Run Mode         : Analysis
Peak Measurement: Peak Area
Calculation Type: Percent

| Peak No. | Peak Name | Result () | Ret. Time (min) | Time Offset (min) | Area (counts) | Sep. Code | Width 1/2 (sec) | Status Codes |
|---|---|---|---|---|---|---|---|---|
| 1 | | 97.2908 | 15.587 | 0.000 | 13660955 | BB | 10.5 | |
| 2 | | 2.7092 | 24.247 | 0.000 | 380412 | BB | 23.2 | |
| | Totals: | 100.0000 | | 0.000 | 14041367 | | | |

Total Unidentified Counts :   14041367 counts

Detected Peaks: 4        Rejected Peaks: 2        Identified Peaks: 0

Multiplier: 1        Divisor: 1        Unidentified Peak Factor: 0

Baseline Offset: -10 microVolts        LSB:    1 microVolts

Noise (used): 52 microVolts - monitored before this run

Manual injection

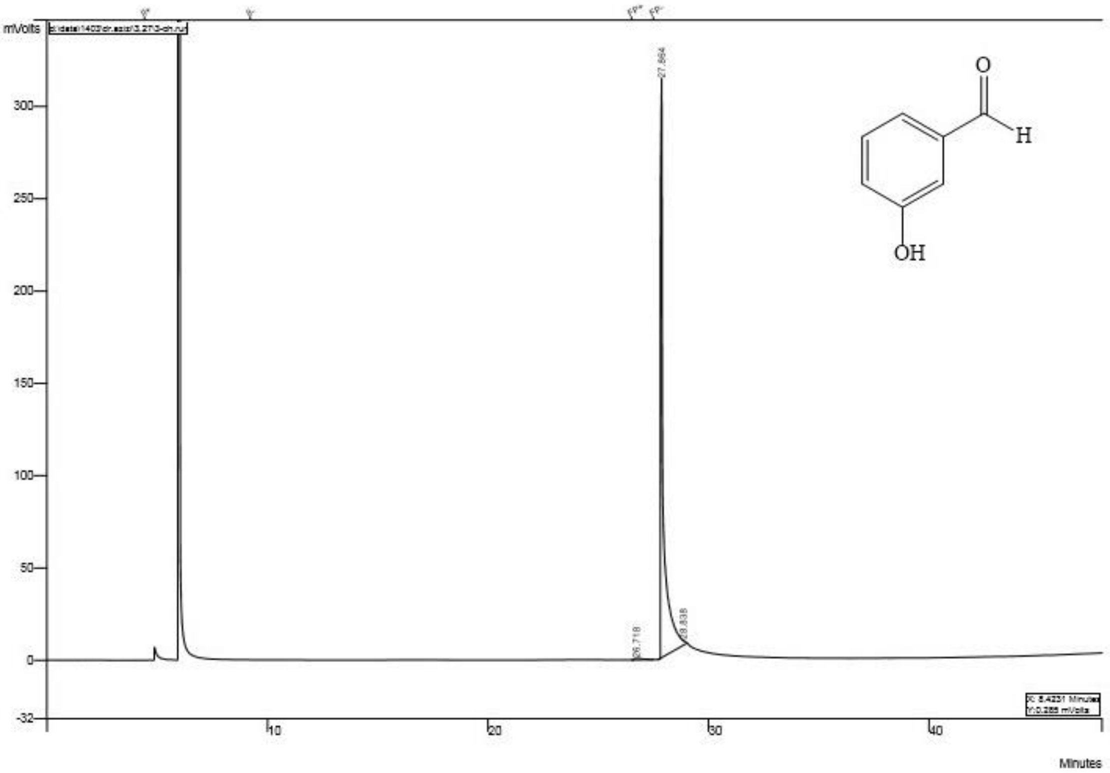


Title        :
Run File     : d:\data\1403\dr.azizi\3.27\3-oh.run
Method File  : 3-oh-middle.mth
Sample ID    : 3-oh

Injection Date: 6/16/2024 12:48 Þ.Ù    Calculation Date: 7/12/2002 06:18 Þ.Ù

Operator    :                          Detector Type: 3800 (100 Volts)
Workstation:                           Bus Address   : 44
Instrument  : Varian Star #1           Sample Rate   : 10.00 Hz
Channel     : Middle = FID             Run Time      : 47.857 min

** GC Workstation Version 6.20 ** 03154-6390-826-0630 **

Run Mode         : Analysis
Peak Measurement: Peak Area
Calculation Type: Percent

| Peak No. | Peak Name | Result () | Ret. Time (min) | Time Offset (min) | Area (counts) | Sep. Code | Width 1/2 (sec) | Status Codes |
|---|---|---|---|---|---|---|---|---|
| 1 | | 0.7767 | 26.718 | 0.000 | 21140 | BB | 29.5 | |
| 2 | | 99.2119 | 27.864 | 0.000 | 2700317 | BB | 4.6 | |
| 3 | | 0.0114 | 28.838 | 0.000 | 309 | TS | 0.0 | |
| | Totals: | 100.0000 | | 0.000 | 2721766 | | | |

Total Unidentified Counts :    2721766 counts

Detected Peaks: 3          Rejected Peaks: 0        Identified Peaks: 0

Multiplier: 1          Divisor: 1          Unidentified Peak Factor: 0

Baseline Offset: 15 microVolts          LSB:     1 microVolts

Noise (used): 70 microVolts - monitored before this run

Manual injection

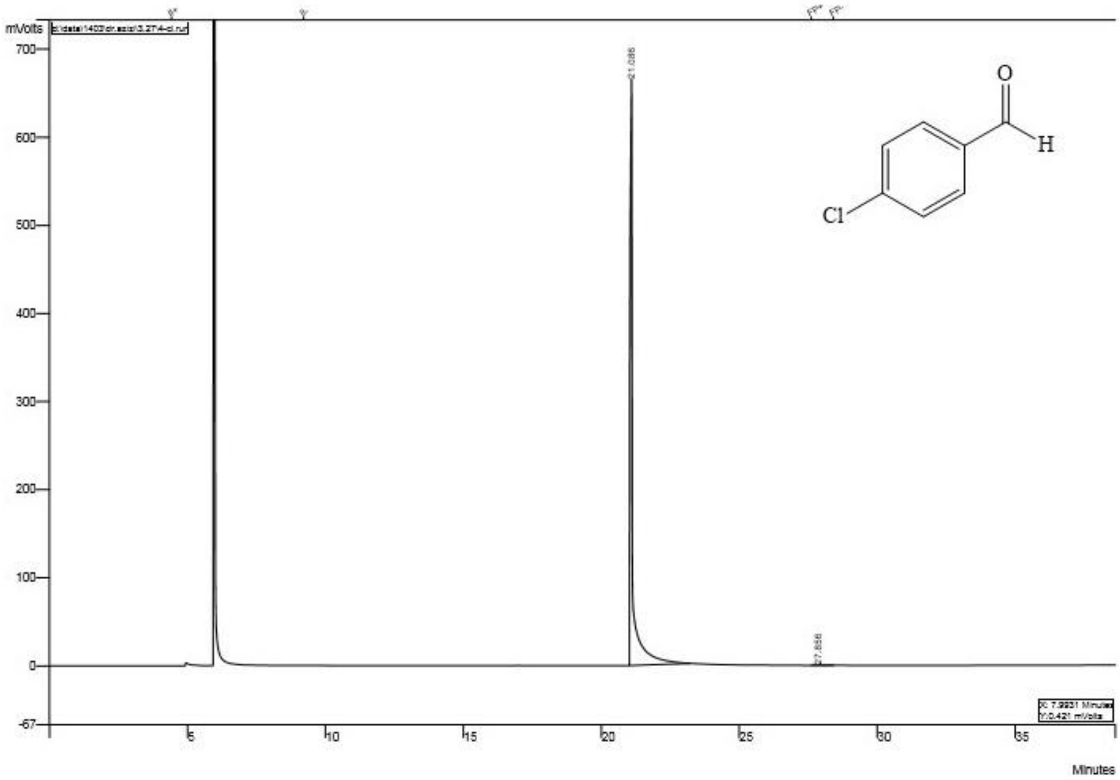


```
Title        :
Run File     : d:\data\1403\dr.azizi\3.27\4-cl.run
Method File  : 4-cl-middle.mth
Sample ID    : 4-cl

Injection Date: 6/16/2024 01:53 Þ.Ù    Calculation Date: 7/12/2002 06:20 Þ.Ù

Operator    :                        Detector Type: 3800 (100 Volts)
Workstation:                         Bus Address   : 44
Instrument  : Varian Star #1         Sample Rate   : 10.00 Hz
Channel     : Middle = FID           Run Time      : 38.642 min

** GC Workstation Version 6.20 ** 03154-6390-826-0630 **

Run Mode          : Analysis
Peak Measurement: Peak Area
Calculation Type: Percent
```

| Peak No. | Peak Name | Result () | Ret. Time (min) | Time Offset (min) | Area (counts) | Sep. Code | Width 1/2 (sec) | Status Codes |
|---|---|---|---|---|---|---|---|---|
| 1 | | 99.9559 | 21.086 | 0.000 | 4431213 | BB | 4.9 | |
| 2 | | 0.0441 | 27.856 | 0.000 | 1956 | BB | 31.6 | |
| | Totals: | 100.0000 | | 0.000 | 4433169 | | | |

```
Total Unidentified Counts :    4433170 counts

Detected Peaks: 2          Rejected Peaks: 0      Identified Peaks: 0

Multiplier: 1          Divisor: 1          Unidentified Peak Factor: 0

Baseline Offset: -3 microVolts          LSB:    1 microVolts

Noise (used): 80 microVolts - monitored before this run

Manual injection
```

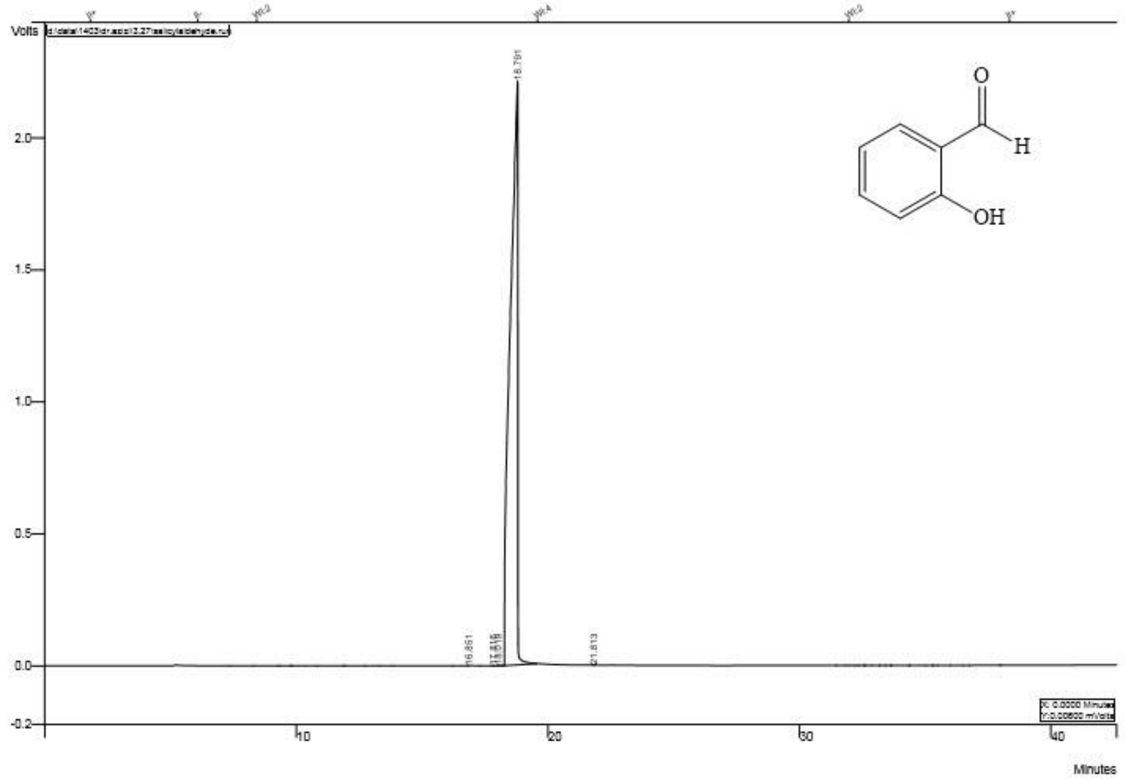


```
Title         :
Run File      : d:\data\1403\dr.azizi\3.27\salicylaldehyde.run
Method File   : salicylaldehyde-middle.mth
Sample ID     : salicylaldehyde

Injection Date: 6/15/2024 11:47 È.Ù     Calculation Date: 7/12/2002 06:21 Þ.Ù

Operator    :                          Detector Type: 3800 (100 Volts)
Workstation:                           Bus Address   : 44
Instrument : Varian Star #1            Sample Rate   : 10.00 Hz
Channel     : Middle = FID             Run Time      : 42.628 min

** GC Workstation Version 6.20 ** 03154-6390-826-0630 **

Run Mode          : Analysis
Peak Measurement: Peak Area
Calculation Type: Percent
```

| Peak No. | Peak Name | Result () | Ret. Time (min) | Time Offset (min) | Area (counts) | Sep. Code | Width 1/2 (sec) | Status Codes |
|---|---|---|---|---|---|---|---|---|
| 1 | | 0.0004 | 16.851 | 0.000 | 162 | BB | 0.0 | |
| 2 | | 0.0012 | 17.816 | 0.000 | 534 | BB | 0.0 | |
| 3 | | 0.0282 | 18.019 | 0.000 | 12670 | BP | 10.6 | |
| 4 | | 99.9698 | 18.791 | 0.000 | 44939104 | PB | 8.6 | |
| 5 | | 0.0005 | 21.813 | 0.000 | 208 | BV | 2.3 | |
| | Totals: | 100.0001 | | 0.000 | 44952678 | | | |

```
Total Unidentified Counts :   44952680 counts

Detected Peaks: 50          Rejected Peaks: 45      Identified Peaks: 0

Multiplier: 1          Divisor: 1          Unidentified Peak Factor: 0

Baseline Offset: 0 microVolts          LSB:     1 microVolts

Noise (used): 33 microVolts - monitored before this run

Manual injection
```

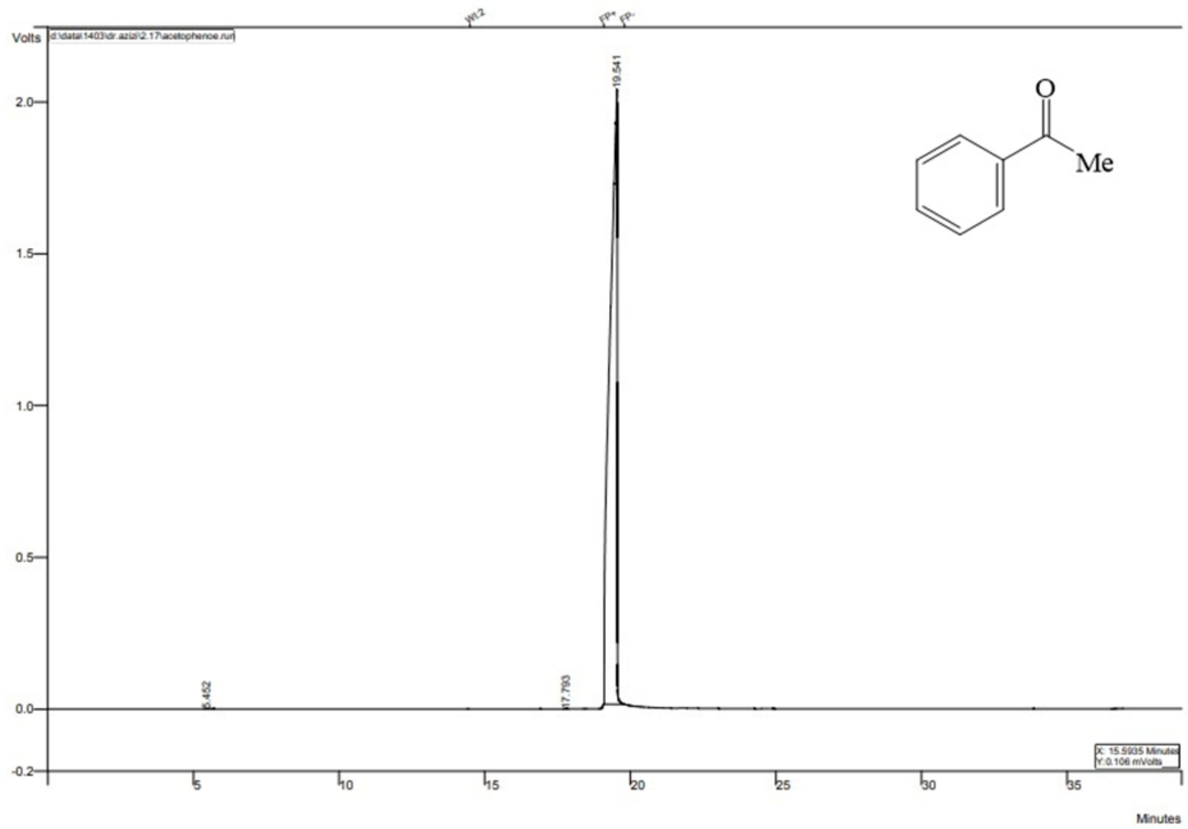


Title :
Run File : d:\data\1403\dr.azizi\2.17\acetophenoe.run
Method File : acetophenoe-middle.mth
Sample ID : acetophenoe

Injection Date: 5/6/2024 11:56 È.Ù Calculation Date: 6/4/2002 05:44 Þ.Ù

Operator :
Workstation:
Instrument : Varian Star #1
Channel : Middle = FID

Detector Type: 3800 (100 Volts)
Bus Address : 44
Sample Rate : 10.00 Hz
Run Time : 38.942 min

** GC Workstation Version 6.20 ** 03154-6390-826-0630 **

Run Mode : Analysis
Peak Measurement: Peak Area
Calculation Type: Percent

| Peak No. | Peak Name | Result () | Ret. Time (min) | Time Offset (min) | Area (counts) | Sep. Code | Width 1/2 (sec) | Status Codes |
|---|---|---|---|---|---|---|---|---|
| 1 | | 0.0060 | 5.452 | 0.000 | 2096 | BB | 0.0 | |
| 2 | | 0.0044 | 17.793 | 0.000 | 1551 | BB | 0.0 | |
| 3 | | 99.9895 | 19.541 | 0.000 | 34891900 | BB | 18.6 | |
| | Totals: | 99.9999 | | 0.000 | 34895547 | | | |

Total Unidentified Counts : 34895548 counts

Detected Peaks: 9 Rejected Peaks: 6 Identified Peaks: 0

Multiplier: 1 Divisor: 1 Unidentified Peak Factor: 0

Baseline Offset: 2 microVolts LSB: 1 microVolts

Noise (used): 38 microVolts - monitored before this run

Manual injection

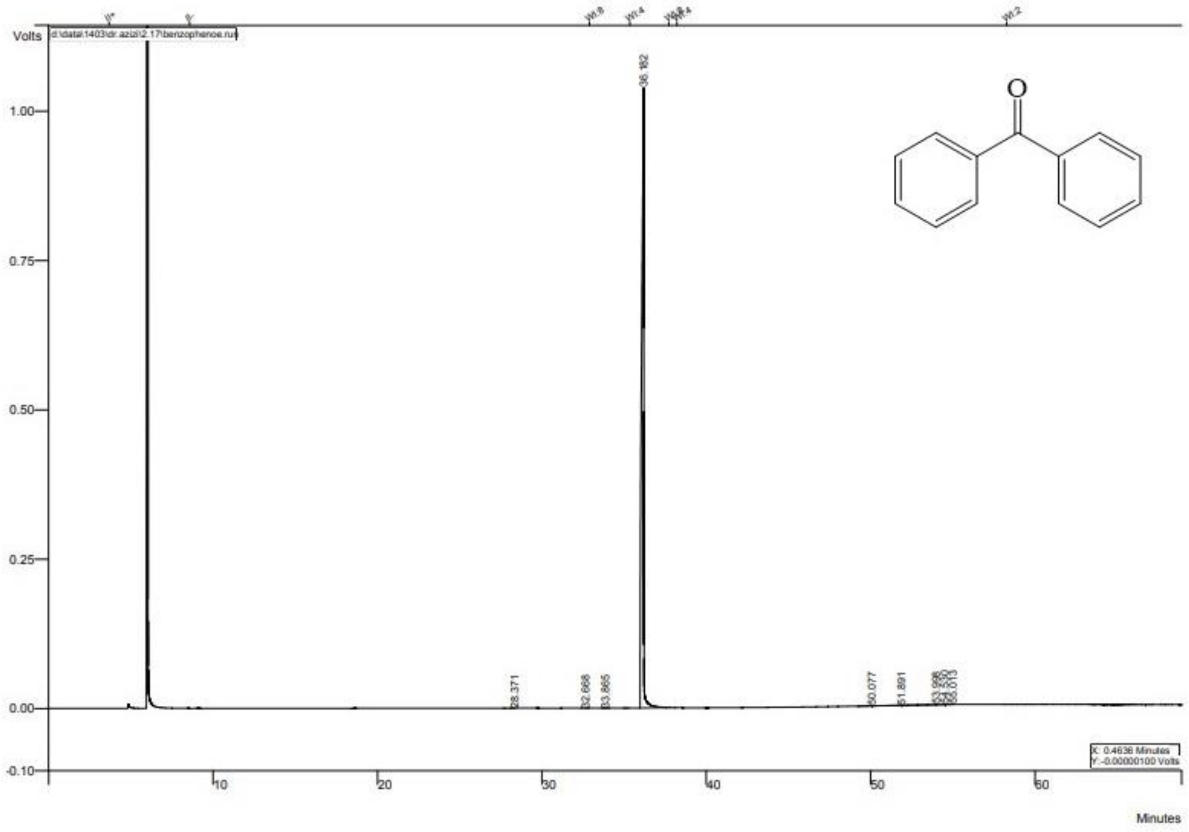

```
Title         :
Run File      : d:\data\1403\dr.azizi\2.17\benzophenoe.run
Method File   : 3-nitrobenzaldehyde-middle.mth
Sample ID     : benzophenoe

Injection Date: 5/7/2024 10:19 È.Ù    Calculation Date: 6/4/2002 05:40 Þ.Ù

Operator    :                         Detector Type: 3800 (100 Volts)
Workstation:                          Bus Address   : 44
Instrument  : Varian Star #1          Sample Rate   : 10.00 Hz
Channel     : Middle = FID            Run Time      : 68.930 min

** GC Workstation Version 6.20 ** 03154-6390-826-0630 **

Run Mode         : Analysis
Peak Measurement: Peak Area
Calculation Type: Percent
```

| Peak No. | Peak Name | Result () | Ret. Time (min) | Time Offset (min) | Area (counts) | Sep. Code | Width 1/2 (sec) | Status Codes |
|---|---|---|---|---|---|---|---|---|
| 1 | | 0.0281 | 28.371 | 0.000 | 2699 | BB | 0.0 | |
| 2 | | 0.0124 | 32.668 | 0.000 | 1188 | BB | 7.6 | |
| 3 | | 0.0342 | 33.865 | 0.000 | 3284 | BB | 0.0 | |
| 4 | | 99.7920 | 36.182 | 0.000 | 9576398 | BB | 9.6 | |
| 5 | | 0.0192 | 50.077 | 0.000 | 1842 | BV | 0.0 | |
| 6 | | 0.0334 | 51.891 | 0.000 | 3206 | VV | 0.0 | |
| 7 | | 0.0387 | 53.998 | 0.000 | 3713 | VV | 0.0 | |
| 8 | | 0.0244 | 54.530 | 0.000 | 2340 | VV | 0.0 | |
| 9 | | 0.0176 | 55.013 | 0.000 | 1685 | VB | 0.0 | |
| | Totals: | 100.0000 | | 0.000 | 9596355 | | | |

```
Total Unidentified Counts :    9596355 counts

Detected Peaks: 69          Rejected Peaks: 60     Identified Peaks: 0

Multiplier: 1          Divisor: 1          Unidentified Peak Factor: 0

Baseline Offset: 2 microVolts          LSB:     1 microVolts

Noise (used): 22 microVolts - monitored before this run

Manual injection
```
